# Effectiveness of Interventions to Improve the Anticholinergic Prescribing Practice in Older Adults: A Systematic Review

**DOI:** 10.3390/jcm11030714

**Published:** 2022-01-28

**Authors:** Mohammed S. Salahudeen, Adel Alfahmi, Anam Farooq, Mehnaz Akhtar, Sana Ajaz, Saud Alotaibi, Manal Faiz, Sheraz Ali

**Affiliations:** 1 School of Pharmacy and Pharmacology, University of Tasmania, Hobart 7001, Australia; adel.alfahmi@utas.edu.au (A.A.); sheraz.ali@utas.edu.au (S.A.); 2 Department of Pharmacy, East Jeddah General Hospital, Ministry of Health, Jeddah 22253, Saudi Arabia; 3 Pharmaceutical Care Department, Dr Sulaiman Al-Habib Hospital, Riyadh 12214, Saudi Arabia; anamfarooq91@gmail.com; 4 Shifa College of Pharmaceutical Sciences, Shifa Tmaeer-e-Millat University, Islamabad 44000, Pakistan; mehnaz.akhtar70@gmail.com (M.A.); sanaajaz.edu@gmail.com (S.A.); 5 Pharmaceutical Care Services, King Saud Medical City, Ministry of Health, Riyadh 12746, Saudi Arabia; saud_2007_sa@hotmail.com; 6 Azra Naheed Medical College, Superior University, Lahore 55150, Pakistan; manalfaiz47@gmail.com

**Keywords:** anticholinergics, intervention, prescribing, older people

## Abstract

Background: Pharmacotherapy in older adults is one of the most challenging aspects of patient care. Older people are prone to drug-related problems such as adverse effects, ineffectiveness, underdosage, overdosage, and drug interactions. Anticholinergic medications are associated with poor outcomes in older patients, and there is no specific intervention strategy for reducing drug burden from anticholinergic activity medications. Little is known about the effectiveness of current interventions that may likely improve the anticholinergic prescribing practice in older adults. Aims: This review seeks to document all types of interventions aiming to reduce anticholinergic prescribing among older adults and assess the current evidence and quality of existing single and combined interventions. Methods: We systematically searched MEDLINE, Embase, Cochrane Central Register of Controlled Trials, CINAHL, and PsycINFO from January 1990 to August 2021. Only studies that examined the effect of interventions in older people focused on improving compliance with anticholinergic prescribing guidelines with quantifiable data were included. The primary outcome of interest was to find the effectiveness of interventions that enhance the anticholinergic prescribing practice in older adults. Results: We screened 3168 records and ended up in 23 studies that met the inclusion criteria. We found only single-component interventions to reduce anticholinergic prescribing errors in older people. Pharmacists implemented interventions without collaboration in nearly half of the studies (*n* = 11). Medication review (43%) and education provision (26%) to healthcare practitioners were the most common interventions. Sixteen studies (70%) reported significant reductions in anticholinergic prescribing errors, whereas seven studies (30%) showed no significant effect. Conclusion: This systematic review suggests that healthcare practitioner-oriented interventions have the potential to reduce the occurrence of anticholinergic prescribing errors in older people. Interventions were primarily effective in reducing the burden of anticholinergic medications and assisting with deprescribing anticholinergic medications in older adults.

## 1. Introduction

Prescribing medications among older adults is recognised as a challenging task and an essential practice that needs to be continuously monitored, assessed, and refined accordingly. Moreover, it is based on understanding clinical pharmacology principles, knowledge about medicines, and particularly the experience and empirical knowledge of the prescribers [1,2]. Clinicians face several challenges while prescribing medications among older adults, and the prescribing of potentially inappropriate medications (PIMs) for this age group is prevalent [3]. The available epidemiological data show that up to 20% of older patients in outpatient settings and 59% of hospitalised older patients consume at least one PIM [4,5,6,7,8]. Adverse effects in older people due to inappropriate prescribing are prevalent, leading to increased hospital admissions and mortality [9].

Medications that possess anticholinergic activity are a class of PIMs widely prescribed for various clinical conditions in older adults [10,11]. Older people are particularly vulnerable to the adverse effects from medicines with anticholinergic-type effects [12,13]. Most medications commonly prescribed to older people are not routinely recognised as having anticholinergic activity, and empirically, clinicians prescribe these medicines based on their anticipated therapeutic benefits while overlooking the risk of cumulative anticholinergic burden [14,15,16]. Anticholinergic burden refers to the cumulative effect of taking multiple medications with anticholinergic activity [17,18]. There is no gold standard approach available to quantify and determine whether an acceptable range of anticholinergic drug burden exists in older adults [19,20]. The central adverse effects of anticholinergic medications are attributed to the excess blocking of cholinergic receptors within the central nervous system (CNS) [16]. The commonly reported central adverse effects are cognitive impairment, headache, reduced cognitive function, anxiety, and behavioural disturbances [16]. The common peripheral adverse effects of anticholinergic medications are hyperthermia, reduced saliva and tear production, urinary retention, constipation, and tachycardia [16].

Anticholinergic medications are associated with poor outcomes in older patients, but there is no specific intervention strategy for reducing anticholinergic drug exposure [21]. There is little evidence that medication review could be a promising strategy in reducing the drug burden in older people [22,23]. Medical practitioner-led and pharmacist-led medication reviews have earlier been reported as a standard practice for reducing anticholinergic drug exposure [24,25]. Pharmacist-led medication review has recently been found to be ineffective among older patients of the Northern Netherlands [25]. A few meta-analyses have also reported the lack of effectiveness of different types of medication reviews on mortality and hospitalisation outcomes [26,27,28]. Multidisciplinary strategies such as patient-centred, pharmacist–physician intervention are also recognised as promising for improving medication use in older patients at risk [29]. Another intervention strategy, i.e., the SÄKLÄK project, had some effects on the PIMs prescription and reduced potential medication-related problems [30]. The SÄKLÄK project is a multi-professional intervention model to improve medication use in older people [30], and it consists of self-assessment using a questionnaire, peer-reviewed by experienced healthcare professionals, feedback report provided by experienced healthcare professionals, and an improvement plan [30].

Interventions to improve prescribing practice more generally have been the subject of many studies and are frequently targeted according to the type of error [31,32]. It is crucial to explore which interventions have effectively changed prescribing practices and optimised patient outcomes while minimising healthcare costs. However, little is known about the effectiveness of existing interventions at improving the anticholinergic prescribing practice for older adults. Hence, this review seeks to document all types of interventions aiming to reduce anticholinergic prescribing errors among older adults and assess the evidence of existing single and combined interventions.

## 2. Methods

The Preferred Reporting Items for Systematic Reviews and Meta-Analyses (PRISMA) guideline was applied to report the findings of this systematic review [33].

### 2.1. Data Sources and Search Strategy

The following databases were examined between January 1990 and August 2021: Ovid MEDLINE, Ovid EMBASE, Ovid PsycINFO, and the Cochrane Central Register of Controlled Trials (CENTRAL). A comprehensive electronic search was performed using appropriate keywords on anticholinergics, older people, and interventions to retrieve the relevant studies. The search was limited to the English language and humans. A detailed MEDLINE search strategy is presented in Supplementary Table S1. Citation analysis was performed in Google Scholar and Web of Science to track the prospective citing of references of the selected articles.

### 2.2. Study Screening and Selection

The title, abstract, and full text of each potentially relevant article were independently screened by two authors (M.S. and S.A.) for eligibility of inclusion in this review. Any discrepancies were resolved by a third author (A.A.), and decisions were made by consensus.

### 2.3. Inclusion Criteria

The primary outcome of interest was single and multicomponent interventions that improve anticholinergic prescribing practice or reduce adverse drug events due to the consumption of anticholinergic medications. Single-component intervention consists of only one intervention activity, such as medication review [34]. Multicomponent intervention refers to the combination of various components in a single intervention [35], such as medication review and the provision of education [34]. All interventions (e.g., medication review, educational detailing visits for physicians, nurses, and aides, pocket-sized educational cards along with clinical vignettes, educational internet site, and detailing session with physicians) performed by any healthcare professional targeting participants of either sex, mean age ≥65 years, and admitted to any healthcare setting were included. We included pre/post or experimental studies that employed a control group.

### 2.4. Exclusion Criteria

We excluded the following studies: review articles, case reports, and case series. We also excluded studies that were conducted in languages other than English.

### 2.5. Data Extraction and Synthesis

**Figure 1:**
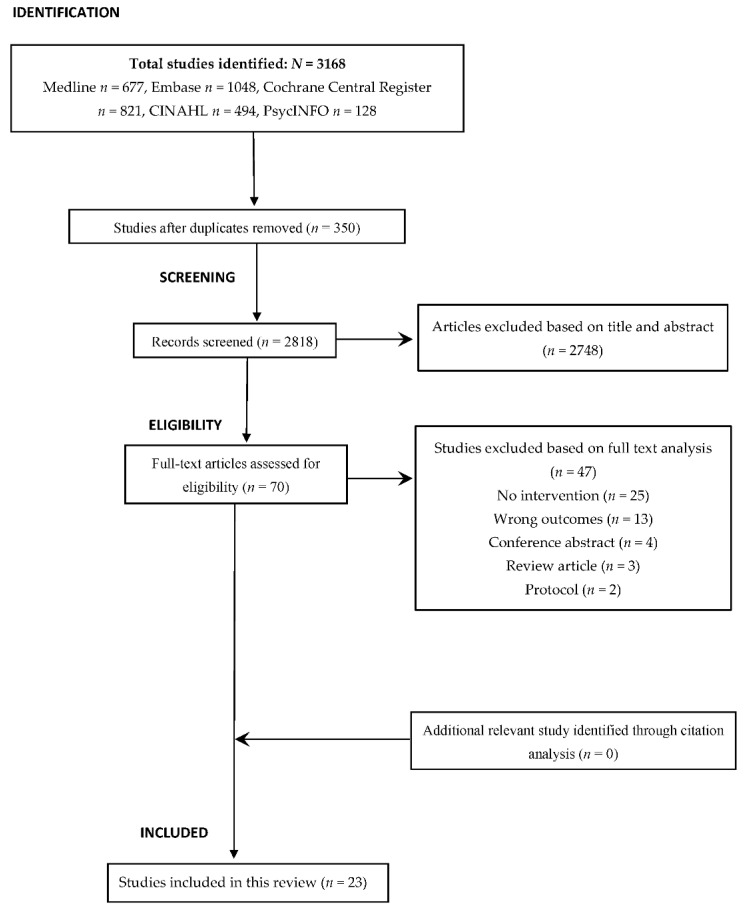
PRISMA flow diagram of the study selection process and citation analysis.

Two reviewers (M.S. and S.A.) independently reviewed and extracted the data from the eligible studies according to a standardised format based on variables of interest, such as the study population, study design and duration, mean age, major findings, and intervention characteristics (type of intervention and implementation). The study selection process is illustrated in Figure 1.

### 2.6. Quality Assessment

The quality of the included studies was critically appraised. The Cochrane Risk of Bias tool [36] was used to assess the methodological quality of the randomised controlled trials (RCTs). The Newcastle-Ottawa scale was used to assess the quality of the non-RCTs [37], which is based on three domains: the selection of study groups, comparability of cohorts and assessment of outcome (cohort studies), or comparability of case and controls and ascertainment of exposure (case-control studies). The thresholds for categorising and interpreting the Newcastle-Ottawa scale domains were described in Supplementary Table S2. The Cochrane Risk of Bias tool results for included RCTs were described in Supplementary Table S3. Studies were not excluded based on the risk of bias or quality assessment.

## 3. Results

The primary electronic search identified a total of 3168 studies from the five databases. Using EndNote X9 (Thomson Reuters), we eliminated 350 duplicate studies, and the remaining 2818 studies were examined to determine their relevance for inclusion. Of those, only 70 were found to be eligible for full-text analysis. Subsequently, 47 studies were excluded as they failed to meet the predefined inclusion criteria. No potential studies were identified from the citation analysis. Finally, a total of 23 studies that investigated the effectiveness of anticholinergic prescribing practice in older adults were included in this review (Figure 1).

### 3.1. Overview of the Included Studies

Table 1 provides the qualitative summary of the included studies, mainly showcasing the type of interventions, and Table 2 illustrates an overview of the quantitative summary of the studies based on study design, setting, sample size, study duration and follow-up, outcome measure (control/pre and intervention/post), significant association (+ or −), and statistical tests.

The countries of origin were USA (*n* = 5) [29,38,39,40,41], Australia (*n* = 4) [22,23,42,43], Finland (*n* = 2) [44,45], Norway (*n* = 2) [21,46], Ireland [47], New Zealand [48], Belgium [49], Spain [50], Sweden [51], Sweden [30], France [52], Italy [53], Taiwan [54], and The Netherlands [55].

The study settings included hospitals (*n* = 7) [40,41,44,46,50,52,53] community/primary care (*n* = 7) [22,30,38,47,49,51,55] and nursing homes/aged care facilities (*n* = 9) [21,23,29,39,42,43,45,48,54]. There were ten cross-sectional studies [22,38,40,42,43,44,46,51,52,54], six nonrandomised or pre/post studies [30,39,47,48,50,53], and seven RCTs [21,23,29,41,45,49,55]. The studies included in this study had sample sizes ranging from 46 to 46,078 study subjects. The average age of the participants varied between 65 and 87.5 years, and the proportion of the female subjects was 39.0–77%.

**Table 1:** The qualitative summary of included studies.

Author, Year, Country	Study Design	Intervention	Description of Intervention(s)	Effect on Outcome/Key Findings
Riordan et al., 2019, Ireland [47]	Convergent parallel mixed-methods design (before and after)	Academic Detailing (pharmacist-led)	Pharmacist conducted face-to-face education sessions and small focus group academic detailing sessions of 19–48 min with physicians.	Pharmacist-led academic detailing intervention was acceptable to GPs. Behavioural Change: awareness of non-pharmacological methods in treating urinary incontinence. Knowledge Gain: intervention served to refresh their knowledge
Ailabouni et al., 2019, New Zealand [48]	A single group (pre-and post-comparison) feasibility study	Medication review (deprescribing)	A collaborative pharmacist-led medication review with GPs was employed. New Zealand registered pharmacists used peer-reviewed deprescribing guidelines. The cumulative use of anticholinergic and sedative medicines for each participant was quantified using the DBI.	Deprescribing resulted in a significant reduction in falls, depression and frailty scores, and adverse drug reactions. No improvement in cognition and quality of life. Total regular medicines use reduced statistically, by a mean difference of 2.13 medicines per patient, among patients where deprescribing was initiated.
Toivo et al., 2019, Belgium [49]	Cluster RCT	Care coordination intervention (coordinated medication risk management)	Practical nurses were trained to make the preliminary medication risk assessment during home visits and report findings to the coordinating pharmacist. The coordinating pharmacist prepared the cases for the triage meeting with the physician and home care nurse to decide further actions.	No significant impact on the medication risks between the intervention and the control group. The per-protocol analysis indicated a tendency for effectiveness, particularly in optimising central nervous system medication use.
Hernandez et al., 2020, Spain [50]	Prospective pre-and post- interventional study	Medication review	Pharmacists reviewed the medications and detected drug-related problems using the Drug Burden Index (DBI) tool. Their recommendations were communicated to the physician via telephone, weekly meetings, and email. Further review was conducted at the weekly meeting between physician and pharmacist.	Statistically significant differences were found between pre- and post-intervention in NPI at admission, drug-related problems, MAI criteria (interactions, dosage and duplication), and mean (SD) DBI score.
Lenander et al., 2018, Sweden [51]	Cross-sectional	Medication Review	Clinical Pharmacist led medication review to assess the prevalence of DRPs and recommendations to discontinue, followed by team-based discussions with general practitioners (GPs) and nurses	It shows that the medication reviews decreased the use of potentially inappropriate medication.
Weichert et al., 2018, Finland [44]	Multicentre observational study	Medication Review	Medication review was conducted for ACB in patients at the time of admission and discharge	21.1% of patients had their ACB reduced. There is considerable scope for improvement of prescribing practices in older people.
Lenander et al., 2017, Sweden [30]	Interventional pilot study	SÄKLÄK project, a developed intervention model	Multi-professional intervention model created to improve medication safety for elderly	Significant decrease in the prescription of anticholinergic drugs indicated the SÄKLÄK intervention is effective in reducing potential DRPs
Moga et al., 2017, USA [29]	Parallel arm Randomised Interventional study	Targeted medication therapy management intervention	Targeted patient-centred pharmacist–physician team medication therapy management intervention was used to reduce the use of inappropriate anticholinergic medications in older patients.	The targeted medication therapy management intervention resulted in improvement in anticholinergic medication appropriateness and reduced the use of inappropriate anticholinergic medications in older patients.
Lagrange et al., 2017, France [52]	Retrospective study	A context-aware pharmaceutical analysis tool	A context-aware computerised decision-support system designed to automatically compare prescriptions recorded in computerised patient files against the main consensual guidelines for medical management in older adults.	Prescription of anticholinergics was significantly decreased (28%).
Carnahan et al., 2017, USA [39]	Quasi-experimental study design	Educational program on medication use	IA-ADAPT/CMS Partnership is an evidence-based training program to improve dispensing drugs for elderly	Suggests that the IA-ADAPT and the CMS Partnership improved medication use with no adverse impact on BPSD.
Hanus et al., 2016, USA [40]	Observational Pilot study	Pharmacist-led EHR-based population health initiative and ARS Service	Physicians in the primary care settings could communicate with pharmacists employing a shared EHR. As part of a quality improvement project, a pharmacist-led EHR-based medication therapy recommendation service was implemented at 2 DHS medical clinics to reduce the anticholinergic burden	High recommendation acceptance rates were achieved using objective anticholinergic risk assessment and algorithm-driven medication therapy recommendations.
McLarin et al., 2016, Australia [43]	Retrospective study	RMMR	Impact of RMMRs on anticholinergic burden quantified by seven anticholinergic risk scales	Demonstrated that RMMRs are effective in reducing ACM prescribing in elderly
Kersten et al., 2015, Norway [46]	Retrospective study	Medication review	Investigated the clinical impact of PIMs in acutely hospitalised older adults.	Anticholinergic prescriptions were reduced from 39.2% to 37.9%
Juola et al., 2015, Finland [45]	Cluster RCT	Educational intervention	Nursing staff working in the intervention wards received two 4-h interactive training sessions based on constructive learning theory to recognise harmful medications and adverse drug events.	No significant differences in the change in prevalence of anticholinergic drugs.
Kersten et al., 2013, Norway [21]	RCT	Multidisciplinary drug review	Single Blind MDRD was conducted that recruited long-term nursing home residents with a total ADS score of greater than or equal to 3	After 8 weeks, the median ADS score was significantly reduced from 4 to 2 in the intervention group. The largest improvement in immediate recall after 8 weeks was observed in the five patients in the intervention group who had their ADS score reduced to 0
Ghibelli et al., 2013, Italy [53]	Pre, post-intervention study	INTERcheck CPSS	INTERcheck is a CPSS developed to optimise drug prescription for older people with multimorbidity and minimise the occurrence of adverse drug reactions.	The use of INTERCheck was associated with a significant reduction in PIMs and new-onset potentially severe DDIs.
Yeh et al., 2013, Taiwan [54]	Prospective case-control study	Educational program for primary care physicians	Educational program for primary care physicians serving in Veterans’ Homes, focusing on anticholinergic adverse reactions in geriatrics and the CR-ACHS	CR-ACHS was significantly reduced in the intervention group at 12-week follow-up.
Boustani et al., 2012, USA [41]	RCT	CDSS Alert (anticholinergic discontinuation)	CDSS alert system sends an interruptive alert if any of the 18 anticholinergics were prescribed, recommending stopping the drug, suggesting an alternative, or recommending dose modification.	Physicians receiving the CDSS issued more discontinuation orders of definite anticholinergics, but the results were not statistically significant. Results suggest that human interaction may play an important role in accepting recommendations aimed at improving the care of hospitalised older adults with CI.
Gnjidic et al., 2010, Australia [23]	Cluster RCT	Medication review	The study intervention included a letter and phone call to GPs, using DBI to prompt them to consider dose reduction or cessation of anticholinergic and sedative medications.	At follow-up, a DBI change was observed in 16 participants. DBI decreased in 12 participants, 6 (19%) in the control group, and 6 (32%) in the intervention group.
Castelino et al., 2010, Australia [22]	Retrospective study	Medication reviews by pharmacist	HMR by pharmacists for leads to an improvement in the use of medications	DBI and PIMs identified in 60.5% and 39.8% of the patients. Significant reduction in the cumulative DBI scores for all patients was observed following pharmacists’ recommendations
Starner et al., 2009, USA [38]	Retrospective study	Educational Intervention	Intervention letters were mailed to the physicians for patients having ≥1 DAE claim	Noticeable decrease was observed after a 6-month follow-up of the intervention in the reduction of DAE claims (48.8%) specifically reduction of anticholinergics (66.7%) was highest
Nishtala et al., 2009, Australia [42]	Retrospective study	RMMR	Clinical Pharmacist-led medication review decreased the DBI in older people	GP’s uptake of recommendations made by pharmacists resulted in a decrease in DBI score. Clinical pharmacist-conducted medication reviews can reduce prescribing of anticholinergic drugs and significantly decrease the DBI score of the study population.
van Eijk et al., 2001, Netherlands [55]	RCT	Educational visits as an individual and a group for general practitioners and pharmacists	Educational visits used academic detailing to discuss prescribing of highly anticholinergic antidepressants in elderly people.	The rate of starting anticholinergic antidepressants in the elderly reduced 26% (in the individual intervention) and 45% (in the group intervention) The use of less anticholinergic antidepressants increased by 40% and 29%, respectively

MAI, medication appropriateness index; GPs, general practitioners; DBI, drug burden index; NPI, neuropsychiatry inventory; RCT, randomised controlled trial; CDSS, clinical decision support system alert; DAE, drugs to be avoided in the elderly; DRPs, drug-related problems; CI, cognitive impairment; ACB, anticholinergic burden; MDRD, modification of diet in renal disease study equation; ADS, anticholinergic drug scale; PIMs, potentially inappropriate medications; CPSS, computerised prescription support system; DDIs, drug–drug interactions; EHR, electronic health record; DHS, Department of Health Services; ARS, anticholinergic risk scale; CR-ACHS, clinician-rated anticholinergic score; HMR, home medicines review; IA-ADAPT, improving antipsychotic appropriateness in dementia patients; CMS, Centers for Medicare and Medicaid Services Partnership to Improve Dementia Care; BPSD, Behavioural and psychological symptoms of dementia; RMMRs, Residential Medication Management Reviews; ACM, anticholinergic medication.

**Table 2:** The quantitative summary of included studies.

Author, Year, Country	Study Design	Setting	Sample Size	Mean Age (Years)	Gender (Female %)	Study Duration	Follow-Up	Relevant Outcome(s)	Outcome Measure		Significant Association (±)	Statistical Tests
									Control/Pre	Intervention/Post		
Riordan et al., 2019, Ireland [47]	Convergent parallel mixed-methods design (before and after)	General Practice	154	75.0	72.1	5 months	6 months	Effects on DBI and ACB scores	Patients having an ACB score of 0 (34%)	Patients having an ACB score of 0 (31%) 65% of patients did not show any change in DBI over time	−	SD, Range, IQR, Frequency, Percentages
Ailabouni et al., 2019, New Zealand [48]	A single group (pre- and post-comparison) feasibility study	Residential care facilities	46	65.0	74.0	6 months	2 weeks	Reduction in DBI score	≥0.5 (median DBI)	0.34 (median DBI)	+	Wilcox-signed Rank test (WSR) *t*-test Fisher’s exact test
Toivo et al., 2019, Belgium [49]	Cluster RCT	Primary care	129	82.8	69.8	1 year	1 year	Anticholinergic use	18.8% (Anticholinergic use at baseline) 18.8% (Anticholinergic use at 12 months)	29.6% (Anticholinergic use at baseline) 18.5% (Anticholinergic use at 12 months)	−	Binary logistic regression, two-sided statistical tests
Hernandez et al., 2020, Spain [50]	Prospective pre- and post-interventional study	Intermediate care hospital	55	84.6	60.0	12 months	NA	Anticholinergic burden per Drug Burden Index (DBI)	1.38 ± 0.7 (Mean DBI)	1.08 ± 0.7 (Mean DBI)	+	Kolmogorov–Smirnov test Student’s *t*-test
Lenander et al., 2018, Sweden [51]	Cross-sectional	Primary care	1720	87.5	74.5	1 year	8 weeks	Discontinuation of DRPs	Pts with anticholinergics = 9.2%	Pts with anticholinergics = 4.2%	+	Student’s *t*-test, Chi-square
Weichert et al., 2018, Finland [44]	Observational study	Hospital	549	79.6	58.3	1 year, 5 months	30 days	Reduction in ACB Score during the hospital stay	Patients on DAPs on admission = 60.8%	Patients on DAPs on discharge = 57.7	−	Shapiro–Wilk test, Wilcoxon signed-rank test,2 sample *t*-test, Yates and Pearson’s chi-square test multivariate binary logistic regression
Lenander et al., 2017 Sweden [30]	Interventional pilot study	Primary care	2400 to 13,700 patients (estimated)	65–79 (range)	63	9 months	6 months	Reduction in anticholinergic PIMs (before/after)	Anticholinergic prescriptions before intervention (4513)	Anticholinergic prescriptions after intervention (3824)	+	Chi-square test
Moga et al., 2017, USA [29]	Parallel arm Randomised Interventional study	Alzheimer’s Disease Center	49	77.7 ± 6.6	70.0	1 year	8 weeks	Significant reduction in anticholinergic drug scale (ADS) Score	1.0 (0.3)	0.2 (0.3)	+	Student’s *t*-tests (or Wilcoxon rank-sum tests for non-normally distributed variables), Chi-square or Fisher’s exact tests
Lagrange et al., 2017, France [52]	Retrospective study	Hospital	187	73.9	63.1	10.5 months	33 and 37 days	Change in number of prescriptions	6538 doses (Anticholinergics)	4696 doses (Anticholinergics)	+	Descriptive statistics
Carnahan et al., 2017, USA [39]	Quasi-experimental study design	Nursing home	411	86.7	77.0	1 year 9 months	276 days	Anticholinergic use	Mean (SD) 35.9% (12.0%)	Mean (SD) 36.1% (10.9%)	−	Generalised linear mixed logistic regression
								Antipsychotic use	Mean (SD) 17.7% (10.4%)	Mean (SD) 20.7% (10.6%)	+	
Hanus et al., 2016, USA [40]	Observational Pilot study	Medical clinics	59	77 ± 9.3	51.0	2 months	2 weeks	Reduction in ACB Score, Increased medication acceptance rate	1.08 50%	0.89 95%	+	Generalised linear mixed-effects model, paired *t*-test
McLarin et al., 2016, Australia [43]	Retrospective study.	Aged care facilities	814	85.6	69.6	NA	NA	Reduction in anticholinergic medications after a medication review	Mean (SD) 3.73 (1.46)	Mean (SD) 3.32 (1.7)	+	Wilcoxon signed-rank test, ANOVA
Kersten et al., 2015, Norway [46]	Retrospective study	Hospital	232	86.1	59.1	8 months	1 year	Reduction in anticholinergic prescriptions	Prevalence of anticholinergic drugs was significantly reduced (*p* < 0.02)		+	Paired samples Student’s *t*-test, McNamar’s test, Mann–Whitney U tests, ANOVA, linear regression
Juola et al., 2015, Finland [45]	Cluster RCT	Assisted living facilities	227	83.0	70.9	1 year	1 year	Mean Anticholinergic drugs	1.0 (Mean Anticholinergic drugs)	1.2 (Mean Anticholinergic drugs)	−	*t*-tests, Mann–Whitney U tests, or Chi-square tests, GEE models, Poisson regression models
Kersten et al., 2013, Norway [21]	RCT	Nursing home	87	85.0	39.0	8 weeks	8 weeks	Marked reduction in ADS score	Median = 4	Median = 2	+	ANCOVA, Poisson regression analysis
Ghibelli et al., 2013, Italy [53]	Pre- and post-intervention study	Hospital	75 for Pre 75 for Post	81	58.3	4 months	NA	Reduction in ACB score	1.3	1.1	−	Pearson Chi-square test, Student’s *t*-test
Yeh et al., 2013, Taiwan [54]	Prospective case-control	Veteran Home	67	83.4	NA	12 weeks	12 weeks	Anticholinergic Burden (CR-ACHS)	1.0 ± 1.1 (Mean CR-ACHS)	−0.5 ± 1.1 (Mean CR-ACHS)	+	Wilcoxon signed ranks test
Boustani et al.,2012, USA [41]	RCT	Hospital	424	74.8	68.0	21 months	At the time of discharge	Discontinuation of AC prescriptions	anticholinergic discontinued = 31.2%	anticholinergic discontinued = 48.9%	_	Fisher’s exact test, *t*-test, logistic regression, multiple regression
Gnjidic et al., 2010, Australia [23]	Cluster RCT	Self-care retirement village	115	84.3	73.0	13 months	3 months	Drug Burden Index (DBI)	0.26 ± 0.34 (mean DBI)	0.22 ± 0.42 (mean DBI)	−	Kolmogorov–Smirnov test Mann–Whitney nonparametric test X^2^ test
Castelino et al., 2010, Australia [22]	A retrospective analysis of medication reviews	Community-dwelling	372	76.1	55.0	NA	NA	Impact of pharmacist’s on DBI scores	Sum of DBI scores = 206.86	Sum of DBI scores = 157.26	+	Wilcoxon signed-rank test
Starner et al., 2009, USA [38]	Retrospective study	Pharmacy claims data	10,364	65.0	NA	8 months	6 months	Rate of discontinued anticholinergics	NA	66.7%	+	NA
Nishtala et al., 2009, Australia [42]	Retrospective study	Aged care homes	500	84.0	75.0	6 months	2 months	Significant decrease in DBI score	NA	12% decrease in DBI	+	2-tailed Wilcoxon signed-rank test
van Eijk et al., 2001, Netherlands [55]	RCT	Primary care	46,078	71	58.0	1 year	NA	Reduction in the prescribing of anticholinergics	30% reduction in the rate of starting highly anticholinergic antidepressant in the individual intervention arms compared with the control arm	40% reduction in the rate of starting highly anticholinergic antidepressants in the group intervention arms compared with the control arm	+	Poisson regression model

RCT, randomised controlled trial; DBI, drug burden index; DRPs, drug-related problems; ACB, anticholinergic burden; PIM, potentially inappropriate medications; WSR, Wilcox-signed rank test; SD, standard deviation; GPs, general practitioner; CR-ACHS, clinician-rated anticholinergic score; and NA, not available.

### 3.2. Methodological Quality of Studies

All eligible studies were rated for their methodological quality, and many studies (*n* = 14, 61%) were identified to be of good quality based on the Newcastle-Ottawa scale [22,30,38,39,42,43,44,46,47,48,50,51,52,53] (Table S2). The quality of the RCTs was critically appraised using the Cochrane risk of bias assessment tool as shown in Supplementary Table S3. There was a general lack of adequate blinding between study subjects and healthcare practitioners, and between outcomes and assessors. Nonetheless, the follow-up duration was either not clearly specified or insufficient (less than six months) in many studies [21,22,23,29,40,41,42,43,44,48,50,51,52,53,54,55]. Altogether, the studies had a duration of follow-up ranging from 14 days [40,48] to 1 year [45,46,49] (Table 2).

### 3.3. Intervention Characteristics

All studies tested single-component interventions, and medication review was the most common single-component healthcare practitioner-oriented intervention [21,22,23,42,43,44,46,48,50,51,54] followed by the provision of education to the healthcare practitioners [38,39,45,47,54,55]. Healthcare practitioners conducted medication reviews using patient notes or tools such as drug burden index (DBI) and anticholinergic burden (ACB) [23,42,43,44,48,50]. Pharmacists implemented interventions without collaboration with other healthcare practitioners in nearly half of the studies (*n* = 11).

Healthcare practitioner-initiated education mainly consisted of professional components, such as academic detailing sessions for physicians [47,54,55], evidence-based training programs to improve dispensing [39], interactive training sessions for nurses [45], and mailing of intervention letters to the physicians [38]. In three studies [21,29,30], healthcare practitioners also performed interventions such as targeted patient-centred, pharmacist–physician team medication therapy management (MTM) intervention, SÄKLÄK project, and multidisciplinary medication review in collaborations with other healthcare practitioners. A context-aware pharmaceutical analysis tool was tested in France to automatically compare prescriptions recorded in computerised patient files against the main consensual guidelines [52]. Another study tested the clinical decision support system to discontinue orders of definite anticholinergic medications for hospitalised patients with cognitive impairment [41]. Similarly, a study tested targeted patient-centred pharmacist–physician team MTM intervention to reduce the consumption of inappropriate anticholinergic medications in older patients [29]. In Italy, researchers tested the INTERcheck computerised prescription support system to optimise drug prescriptions and minimise the occurrence of adverse drug reactions [53].

### 3.4. Effectiveness of Interventions at Improving Anticholinergic Prescribing Practice

Sixteen studies (70%) [21,22,29,30,38,39,40,42,43,46,48,50,51,52,54,55] investigating a healthcare practitioner-oriented intervention reported a significant reduction in anticholinergic prescribing errors, whereas seven studies (30%) [23,41,44,45,47,49,53] reported no significant effect (Table 2). Similarly, medication review (*n* = 8) and the provision of education (*n* = 4) were the most common interventions in these sixteen studies; however, these studies varied in their designs. There were 14 studies (87.5%) [22,30,38,39,42,43,44,46,47,48,50,51,52,53] that were of high quality, and of those, 11 studies [22,30,38,39,42,43,46,48,50,51,52] showed a significant reduction in anticholinergic prescribing errors. Seven studies had a follow-up period of ≥6 months, and four studies showed a significant reduction in anticholinergic prescribing errors. With a shorter follow-up period of 2 weeks to 6 months, 4 studies [42,48,51,52] out of 10 studies reported reductions in anticholinergic prescribing errors (Table 2).

Healthcare practitioner-oriented interventions that reported a significant reduction in anticholinergic prescribing errors included: medication review, education provision to healthcare practitioners, pharmacist-led electronic health record-based population health initiative and anticholinergic risk scale service, targeted patient-centred, pharmacist–physician team MTM intervention, context-aware pharmaceutical analysis tool, and SÄKLÄK project. Healthcare practitioner-oriented interventions were most effective in reducing ACB [21,29,40,54], DBI [22,42,48,50], and discontinuation or reduction of anticholinergic medications [30,38,39,43,46,51,52,55]. Hernandez et al. 2020 reported a decline in DBI from 1.38 (control group) to 1.08 (intervention group) [50]. Another study reported a reduction in ACB score from 1.08 (control group) to 0.89 (intervention group) [40]. A retrospective study by McLarin et al. [43] in Australia found a reduction in the mean scores of anticholinergic medications from 3.73 to 3.02 after implementing medication review.

## 4. Discussion

This is believed to be the first systematic review assessing the effectiveness of interventions to reduce anticholinergic prescribing errors in adults aged 65 and above. Previous reviews primarily evaluated the studies of pharmacist-oriented interventions on medication prescribing and the association between anticholinergic drug burden and mortality in older people [17,56,57]. We did not conduct a meta-analysis because of the methodological heterogeneity between the study designs, anticholinergic prescribing errors, types of interventions, study duration, and follow-up period. Given the high prevalence of inappropriate prescribing and polypharmacy in older people aged 65 and over, interventions to reduce anticholinergic prescribing errors in this cohort are of considerable importance. This systematic review identified 23 studies reporting interventions to reduce anticholinergic prescribing errors in older people. The interventions were mainly provided by the pharmacists using a patient-centred approach. Many studies (19 out of 23) successfully reduced the incidence of anticholinergic prescribing errors in older people. Evidence related to the pharmacist-led interventions in many studies suggests that pharmacists play a vital role in the care of older people, thus improving medication safety across the continuum of care.

In this study, medication review and education provision to the healthcare practitioners were the most common elements in many interventions. Medication review is a structured evaluation of patients’ pharmacotherapy to optimise drug use and reduce the occurrence of drug-related problems [58]. Similarly, medication review is recognised as an important healthcare practitioner-oriented intervention for reducing anticholinergic prescribing errors in older people [59]. Likewise, older people benefit mostly from medication reviews as this cohort is more susceptible to adverse drug effects [60,61]. The efficacy of medication review in reducing anticholinergic prescribing errors was reported by eight studies in this review [15,21,22,43,44,46,50,62]. Previous studies inform the significant effects of structured medication review on medication prescriptions and older adults’ quality of life [63,64,65].

Another intervention, such as the provision of education to the healthcare practitioners, was tested in eight studies, but only five studies reported the effectiveness of this intervention in reducing anticholinergic prescribing errors in older people [38,49,54,55,66]. Evidence informs that the healthcare practitioner-oriented educational intervention effectively reduces prescribing errors in older people [57,67]. The provision of education reduces the use of healthcare resources, including emergency department presentations and hospital admissions [68]. Implementing healthcare practitioner-led educational interventions encourages prescribers to change prescription practices, thus improving prescribers’ clinical practice [69]. An education intervention provides precise knowledge about prescribing in older adults, medication-related errors, and prevention strategies for reducing medication-related errors [69]. This review also showed that interventions such as INTERcheck, SÄKLÄK intervention model, targeted MTM intervention, context-aware pharmaceutical analysis tool, and CDSS alert were not successful in reducing anticholinergic prescribing errors in older adults [29,41,51,52,53].

### 4.1. Implications for Clinical Practice and Future Research

Medications with anticholinergic activity are frequently prescribed in older people due to their numerous clinical benefits; however, these medications are also associated with poor clinical outcomes [70]. Implementing healthcare practitioner-oriented interventions can reduce the occurrence of anticholinergic prescribing errors in older people. This review’s findings inform that healthcare practitioner-oriented interventions appear to improve medication safety in older people based on observed reductions in anticholinergic prescribing errors, particularly when the provision of care involves a medication review and an education for the physicians prescribing anticholinergic medications. The prescribing competency can be optimised through educational interventions [71], thus reducing the occurrence of anticholinergic prescribing errors. The safe and effective prescribing of medications is a challenge in older people who frequently experience multiple long-term conditions and complex polypharmacy [72]. Due to an increasing challenge to physicians when prescribing and the complexity of medication regimens taken by older people, there is a need to embed prescribing competency framework in clinical practice [72]. The prescribing competency framework engages prescribers in different stages of prescribing, such as information gathering, clinical decision making, communication, monitoring, and review [72].

The current quantification methods for anticholinergic burden tend to streamline the complexity of pharmacological mechanisms in geriatric risk assessment in older adults. However, there is no universally accepted quantification method available to estimate anticholinergic drug burden, and it is difficult to compare the study findings from distinct methods [16]. Existing tools derived from expert consensus limit the quantification of anticholinergic burden as they do not take into consideration the dose and the CNS distribution of drugs [15]. A recent review showed that the ratings of anticholinergic activity in the expert opinion scales were inconsistent [73]. Moreover, the estimation of central cognitive effects by measuring in vitro serum assay of medications with known anticholinergic activity as a composite peripheral measure still remains unclear [16]. The lack of a gold standard method for anticholinergic quantification might have a direct or indirect impact on the interpretation of the effect size of the study interventions (e.g., reduction in anticholinergic burden). In this review, many studies (69%) were either cross-sectional or nonrandomised and included a single-component intervention. Therefore, there is a need to conduct future randomised multicomponent intervention trials for evaluating the true impact of healthcare practitioner-oriented interventions on anticholinergic prescribing errors in older people.

### 4.2. Strengths and Limitations

This study was comprehensive in that the electronic search, conducted in four important databases, attempted to identify the complete existing body of evidence of the effectiveness of healthcare-oriented interventions aimed at reducing anticholinergic prescribing errors in older people. This is the first review that found 17 different types of healthcare-oriented interventions, and their impact on anticholinergic prescribing errors. A limitation of this study included the absence of meta-analysis and the estimation of the effect size. It was mainly due to the heterogeneity of included studies. We also excluded studies published in languages other than English, which may have introduced a language bias. However, we performed citation tracking and hand-searching of all included studies to minimise the influence of factors (e.g., inconsistent terminology or wrong indexing) that may affect the keyword-based search.

## 5. Conclusions

This systematic review suggests that healthcare practitioner-oriented interventions have the potential to reduce the occurrence of anticholinergic prescribing errors in older people. Medication review and the provision of education to the prescribers were the most common approaches to reducing anticholinergic prescribing errors in older people. Healthcare practitioner-oriented interventions were mostly effective in reducing the burden of anticholinergic medications and facilitating the discontinuation of anticholinergic medications in older people. In the future, there is also the need to ascertain how often the healthcare practitioner performs interventions that may reduce anticholinergic prescribing errors in older people.

## Data Availability

All research data generated or analysed during this review are included in this published article.

## Supplementary Materials

The following supporting information can be downloaded at: https://www.mdpi.com/article/10.3390/jcm11030714/s1, Table S1: MEDLINE search strategy; Table S2: The Newcastle-Ottawa scale risk of bias assessment for included cohort studies; Table S3: The Cochrane Risk of Bias tool results for included RCTs.
