# Supplementary material for: Effectiveness of Interventions to Improve the Anticholinergic Prescribing Practice in Older Adults: A Systematic Review
Source: J Clin Med. 2022 Jan 28;11(3):714. doi: 10.3390/jcm11030714 (PMC8836376; doi:10.3390/jcm11030714)
Supplement: Supplementary file 1 [file jcm-11-00714-s001.zip › jcm-1561171-supplementary.pdf]

**Supplementary Table S1.** MEDLINE search strategy

| Source | Search strategy: keyword/MeSH |
|---|---|
| MEDLINE (Ovid) 1990 to August 2021 | 1. Anticholinergic.mp. or exp Cholinergic Antagonists/ (90034)<br>2. anticholinergic*.mp. (12552)<br>3 antimuscarinic#.mp. (773)<br>4 Anticholinergic prescrib*.mp. (8)<br>5 Muscarinic Antagonists.mp. or exp Muscarinic Antagonists/ (55192)<br>6 Cholinergic Antagonists.mp. or exp Cholinergic Antagonists/ (84271)<br>7 Parasympatholytics.mp. or exp Parasympatholytics/ (44696)<br>8 cholinolytic*.mp. (598)<br>9 Antimuscarinic prescrib*.mp. (2)<br>10 1 or 2 or 3 or 4 or 5 or 6 or 7 or 8 or 9 (102500)<br>11 intervention.mp. or exp Early Intervention, Educational/ or exp Early Medical Intervention/ (662546)<br>12 Collaboration.mp. (80214)<br>13 exp "Drug Utilization Review"/ or exp Medication Therapy Management/ or exp "Drug-Related Side Effects and Adverse Reactions"/ or Medication review.mp. or exp Medication Reconciliation/ (128503)<br>14 exp Medical History Taking/ or exp Medication Errors/ or Medication history.mp. (40748)<br>15 Integrated medicine management.mp. (2)<br>16 Outreach Visi*.mp. (303)<br>17 detailing.mp. (7444)<br>18 Partnered.mp. (3137)<br>19 Charting.mp. (3347)<br>20 Adverse drug event.mp. (1109)<br>21 ADE.mp. (2684)<br>22 Medication safety.mp. (2533)<br>23 drug safety.mp. (5581)<br>24 Medication appropriateness.mp. (243)<br>25 Inappropriate medi#.mp. (10)<br>26 Inappropriate drug.mp. (527)<br>27 Prescribing.mp. (50108) |

| | | |
|---|---|---|
| | 28 | 11 or 12 or 13 or 14 or 15 or 16 or 17 or 18 or 19 or 20 or 21 or 22 or 23 or 24 or 25 or 26 or 27 (953366) |
| | 29 | Aged.mp. or exp "Aged, 80 and over"/ or exp Aged/ (5533046) |
| | 30 | older people.mp. (31764) |
| | 31 | elderly.mp. (267270) |
| | 32 | older adul#.mp. (8550) |
| | 33 | geriatric*.mp. (107308) |
| | 34 | 29 or 30 or 31 or 32 or 33 (5620543) |
| | 35 | 10 and 28 and 34 (1450) |
| | 38 | limit 35 to (english language and humans and yr="1990 -Current") (677) |

**Supplementary Table S2**. The Newcastle-Ottawa scale risk of bias assessment for included cohort studies

| Author, Year, Country | Study design | Selection | Comparability | Outcome |
|---|---|---|---|---|
| Riordan et al., 2019, Ireland **(46)** | A single group (pre- and post-comparison) feasibility study | ☆☆☆ | ☆☆ | ☆☆ |
| Ailabouni et al.,2019, New Zealand **(47)** | Convergent parallel mixed methods design (before and after) | ☆☆☆ | ☆☆ | ☆☆ |
| Hernandez et al., 2020, Spain **(49)** | Prospective pre-post interventional study | ☆☆☆ | ☆☆ | ☆☆ |
| Lenander et al.,2018, Sweden **(50)** | Cross-sectional | ☆☆☆☆ | ☆☆ | ☆☆☆ |
| Weichert et al.,2018, Finland **(42)** | Multi-centre observational study | ☆☆☆☆ | ☆☆ | ☆☆☆ |
| Lenander et al, 2017, Sweden **(28)** | Interventional pilot study | ☆☆☆☆ | ☆☆ | ☆☆☆ |
| Lagrange et al., 2017, France **(51)** | Retrospective study | ☆☆☆ | ☆☆ | ☆☆☆ |

| | | | | |
|---|---|---|---|---|
| Carnahan et al., 2017, US **(37)** | Quasi-experimental study design | ☆☆☆☆ | ☆☆ | ☆☆☆ |
| Hanus et al., 2016, US **(38)** | Observational Pilot study | ☆☆ | ☆☆ | ☆☆ |
| McLarin et al, 2016, Australia **(41)** | Retrospective study | ☆☆☆☆ | ☆☆ | ☆ |
| Kersten et al.,2015, Norway **(44)** | Retrospective study | ☆☆☆☆ | ☆☆ | ☆☆☆ |
| Ghibelli et al., 2013, Italy **(52)** | Pre, post study | ☆☆☆ | ☆☆ | ☆☆ |
| Yeh et al., 2013, Taiwan **(53)** | Prospective case–control study | ☆☆ | ☆☆ | ☆☆☆ |
| Castelino et al , 2010, Australia **(20)** | Retrospective study | ☆☆☆☆ | ☆☆ | ☆ |
| Nishtala et al.,2009, Australia **(40)** | Retrospective study | ☆☆☆☆ | ☆☆ | ☆☆☆ |
| Starner et al., 2009, USA **(36)** | Retrospective study | ☆☆☆☆ | ☆☆ | ☆☆☆ |

**Note**: A study can be given a maximum of one star for each numbered item within the *Selection* (maximum of 4 stars)) and *Outcome* categories (maximum of 3 stars). A maximum of two stars can be given for *Comparability*.

Thresholds for converting the Newcastle-Ottawa scale to Agency for Healthcare Research and Quality (AHRQ) standards (good, fair, and poor):

*Good quality*: 3 or 4 stars in selection domain AND 1 or 2 stars in comparability domain AND 2 or 3 stars in outcome/exposure domain

*Fair quality*: 2 stars in selection domain AND 1 or 2 stars in comparability domain AND 2 or 3 stars in outcome/exposure domain

*Poor quality*: 0 or 1 star in selection domain OR 0 stars in comparability domain OR 0 or 1 stars in outcome/exposure domain.

**Supplementary Table S3.** The Cochrane Risk of Bias tool results for included RCTs

| Study | Study design | Randomisation sequence generation | Allocation concealment | Blinding of participants and personnel | Blinding outcome assessors | Loss to follow up (incomplete data) | Selective reporting |
|---|---|---|---|---|---|---|---|
| Kersten et al.,2013, Norway (45) | RCT, single-blinded | ✓ | X | X | ? | X | ✓ |
| van Eijk et al, 2001, Netherlands (54) | RCT | X | ✓ | X | X | ? | ✓ |
| Boustani et al.,2012, US (39) | RCT | ✓ | ? | X | X | ✓ | ✓ |
| Juola et al., 2015, Finland (43) | Cluster RCT | ✓ | ? | X | X | ✓ | ✓ |
| Toivo et al., 2019, Belgium (48) | Two-arm, parallel, cluster RCT | ? | ? | X | X | ✓ | ✓ |
| Moga et al., 2017, USA (27) | Parallel arm RCT | ✓ | ✓ | X | X | ✓ | ✓ |
| Gnjidic et al., 2010, Australia (21) | Cluster RCT | ✓ | ✓ | X | X | ✓ | ✓ |

X = inadequate; ✓ = adequate; ? = unclear

RCT = Randomised controlled trial
